# Bi-Directional Communication Between Neurons and Astrocytes Modulates Spinal Motor Circuits

**DOI:** 10.3389/fncel.2020.00030

**Published:** 2020-02-27

**Authors:** Matthew J. Broadhead, Gareth B. Miles

**Affiliations:** School of Psychology and Neuroscience, University of St Andrews, St Andrews, United Kingdom

**Keywords:** astrocyte, spinal cord, locomotion, neuromodulation, mGlu receptor5

## Abstract

Evidence suggests that astrocytes are not merely supportive cells in the nervous system but may actively participate in the control of neural circuits underlying cognition and behavior. In this study, we examined the role of astrocytes within the motor circuitry of the mammalian spinal cord. Pharmacogenetic manipulation of astrocytic activity in isolated spinal cord preparations obtained from neonatal mice revealed astrocyte-derived, adenosinergic modulation of the frequency of rhythmic output generated by the locomotor central pattern generator (CPG) network. Live Ca^2+^ imaging demonstrated increased activity in astrocytes during locomotor-related output and in response to the direct stimulation of spinal neurons. Finally, astrocytes were found to respond to neuronally-derived glutamate in a metabotropic glutamate receptor 5 (mGluR5) dependent manner, which in turn drives astrocytic modulation of the locomotor network. Our work identifies bi-directional signaling mechanisms between neurons and astrocytes underlying modulatory feedback control of motor circuits, which may act to constrain network output within optimal ranges for movement.

## Lay Summary

We have investigated how astrocytes, a type of supportive cell within the nervous system, may be directly involved in the control of movements, such as walking (locomotion). We found that astrocytes are active participants in the neural circuits of the mammalian spinal cord that control locomotion. The function of astrocytes within these networks appears to relate to the modulation of locomotor speed. We also identify the chemical signaling pathways involved in a modulatory feedback loop between astrocytes and neurons. Our findings suggest that this bi-directional communication between astrocytes and neurons may act to constrain spinal motor circuits within operational ranges for desired movements.

## Introduction

Astrocytes, one of the predominant forms of glial cells in the nervous system, are a diverse and multifunctional group of cells with the capacity to integrate numerous neuronal-derived signals and modulate neuronal activity at a network level (Verkhratsky and Butt, 2013). There is growing evidence that astrocytes play a role in a wide range of basic central nervous system (CNS) functions (Gibbs et al., 2008; Robertson, 2018). Within motor systems, astrocytes are implicated in the control of rhythmic behaviors, such as respiration (Gourine et al., 2010; Huxtable et al., 2010; Sheikhbahaei et al., 2018), mastication (Morquette et al., 2015), feeding (Yang et al., 2015; Sweeney et al., 2016) and locomotion (Acton and Miles, 2015, 2017; Witts et al., 2015; Acton et al., 2018). These rhythmic motor behaviors are generated by a dedicated central pattern generator (CPG) networks. For example, the lumbar spinal cord contains a CPG network that consists of interneurons, which generate rhythmic activity, and motor neurons, which convey the network’s output to coordinate alternating left-right and flexor-extensor muscle activation as required for the generation of locomotor behavior (Grillner, 2003, 2006; Selverston, 2005; Kiehn, 2016). CPGs underlying behaviors such as locomotion, respiration, chewing and gut motility, require various modulatory mechanisms to fine-tune the speed, pattern and power of behavior in accordance with the requirements of the animal at any given time (Harris-Warrick, 2011; Miles and Sillar, 2011). Although neurons have traditionally been considered the primary, if not sole, source of such modulation, a growing body of evidence supports important roles for astrocytes in the modulation of CPG networks (Gourine et al., 2010; Acton and Miles, 2015; Morquette et al., 2015; Yang et al., 2015).

Previous research suggests that pharmacological activation or ablation of spinal cord astrocytes alters the synaptic activity of interneurons and modulates the frequency of locomotor output (Witts et al., 2012, 2015; Carlsen and Perrier, 2014; Acton and Miles, 2015; Acton et al., 2018). These effects are thought to be dependent on the astrocytic release of ATP with subsequent conversion to adenosine, which acts *via* neuronal A1 adenosine receptors. Although these findings support that astrocytes are active participants within locomotor circuitry, further analyses utilizing additional methods for activating and, importantly, inhibiting endogenous astrocytic activity are required to provide a greater understanding of their functional roles. Furthermore, if spinal cord astrocytes are indeed active participants in the locomotor CPG, then it is likely their role is driven by neuronally-derived signals, which remain to be identified. Given growing evidence that astrocytes play a role in a host of neurological and neurodegenerative diseases, including chronic pain (Ji et al., 2006; Gao and Ji, 2010), spinal cord injury (Okada et al., 2006) and Amyotrophic Lateral Sclerosis (ALS; Phatnani et al., 2013; Pehar et al., 2018), identifying the physiological roles of astrocytes within spinal circuits, and the signaling mechanisms involved, could pave the way for the development of novel therapeutic strategies.

In this study, we aimed to further our investigation into whether spinal cord astrocytes are active participants within mammalian locomotor circuits and identify the signaling mechanisms between spinal neurons and astrocytes. To address these aims, we have used transgenic mouse lines to both visualize and manipulate astrocytic activity in the isolated mammalian spinal cord *in vitro*. We reveal neuron-astrocyte and astrocyte-neuron signaling mechanisms that underlie modulatory, inhibitory feedback control of motor circuit output.

## Materials and Methods

### Animals

All procedures performed on animals were conducted in accordance with the UK Animals (Scientific Procedures) Act 1986 and were approved by the University of St Andrews Animal Welfare and Ethics Committee. Mice were euthanized *via* cervical dislocation and decapitation. All lines of mice were obtained from Jackson Laboratories and are listed in Table 1.

**Table 1:** Details of the transgenic mouse lines used for the study.

Jackson laboratories stock number	Full name	Abbreviated name	Purpose
012849	*hGFAP::CRE/ERT2*	*GFAP::Cre*	Tamoxifen inducible cre expression in astrocytes
024106	*Ai96(RCL-GCaMP6s)*	*GCAMP6s*	Fluorescent Ca^2+^ indication
026220	*R26-LSL-hM3Dq-DREADD*	*hM3Dq*	Excitatory DREADD receptor
026219	*R26-LSL-hM4Di-DREADD*	*hM4Di*	Inhibitory DREADD receptor
007905	*Gt(ROSA)26Sor*^tm9(CAG-tdTomato)Hze^	*TdTomato*	Fluorescence reporter line

To induce expression of genes in astrocytes, heterozygous GFAP::Cre transgenic mice were injected with 100 μg/g mouse weight of Tamoxifen (Sigma) starting from neonatal day 5 (P5) with subsequent injections every 24 h until sacrificed for the experiment. Experiments requiring ventral root recordings of fictive locomotion in whole isolated spinal cords and hemisected spinal cords of transgenic mice were performed at P6–8. Ca^2+^ imaging experiments were performed on neonates at P6–10. Where transgenic mice were not required for fictive locomotor experiments (with the exception of the non-transgenic controls for DREADD experiments), mice aged P1–5 were used as obtaining fictive locomotion is more reliable in younger mice.

### Tissue Preparation

For ventral root recordings from whole isolated spinal cords or hemisected spinal cords, animals were sacrificed by cervical dislocation, decapitated and eviscerated before being transferred to a dissection chamber containing artificial cerebrospinal fluid (aCSF, equilibrated with 95% O_2_, 5% CO_2_, ~4°C). Spinal cords were isolated between the midthoracic and upper sacral segments, and the dorsal roots trimmed, leaving ventral roots intact. For hemisected spinal cords, the pia was carefully removed and the two halves cleaved gradually with a dissecting pin. For preparing acute spinal cord slices, neonatal mice were sacrificed as described above, and the spinal cord dissected in aCSF. Slices were made at 300 μm thickness from the lumbar segments using a vibratome (VT1200, Leica) and transferred to recovery aCSF maintained at ~34°C (equilibrated with 95% O_2_, and 5% CO_2_) for 30–60 min. Slices were subsequently transferred to recording aCSF.

### Drugs and Solutions

Recording aCSF contained the following (in mM): 127 NaCl, 3 KCl, 1.25 NaH_2_PO_4_, 1 MgCl_2_, 2 CaCl, 26 NaHCO_3_, and 10 glucose. The dissecting aCSF contained the following (in mM): 25 NaCl, 188 sucrose, 1.9 KCl, 1.2 NaH_2_PO_4_, 10 MgSO_4_, 1 CaCl, 26 NaHCO_3_, 25 glucose, and 1.5 kynurenic acid. The recovery aCSF contained the following (in mM): 119 NaCl, 1.9 KCl, 1.2 NaH_2_PO_4_, 10 MgSO_4_, 1 CaCl, 26 NaHCO_3_, 20 glucose, and 1.5 kynurenic acid. Intracellular solution used for single-cell recordings contained the following (in mM): 140 KMeSO_4_, 10 NaCl, 1 CaCl, 10 HEPES, 1 EGTA, 3 Mg-ATP, and 0.4 GTP-Na_2_ (pH 7.2–7.3, adjusted with KOH).

The following drugs were purchased from Sigma: Glutamate, γ-aminobutyric acid (GABA), N-methyl-D-aspartate (NMDA) and Dopamine (DA). The following drugs were purchased from Tocris: 8-Cyclopentyl-1,3-dipropylxanthine (DPCPX), 2-Methyl-6-(phenylethynyl)pyridine hydrochloride (MPEP), 5-hydroxytryptamine (5-HT) and (RS)-2-Chloro-5-hydroxyphenylglycine (CHPG). Tetrodotoxin (TTX) and CNO were purchased from HelloBio. With the exception of Glutamate, CHPG, MPEP and DPCPX, all drugs were made up in deionized H_2_O. Glutamate and CHPG were made up in 1 M NaOH, while MPEP and DPCPX were made up in dimethyl sulfoxide (DMSO). CNO and DPCPX were prepared fresh on a weekly basis. Drug concentrations were chosen based on previous publications (Bowman and Kimelberg, 1984; Queiroz et al., 1999; Parri et al., 2010; Iwagaki and Miles, 2011; Takeuchi et al., 2014; Acton and Miles, 2015; Mariotti et al., 2016; Jennings et al., 2017).

### Ca^2+^ Imaging

Ca^2+^ imaging of spinal cord slices was performed in aCSF (equilibrated with 95% O_2_, 5% CO_2_) warmed to ~34°C with an inline heater (Warner Instruments). Imaging of hemisected spinal cords was performed at 20–24°C in order to ensure robust fictive locomotor output. Images were captured as described in Acton et al. (2018). Briefly, images were acquired using a Zyla 4.2 scientific CMOS camera (Andor, Oxford Instruments) using a 40× water immersion objective lens (0.9 numerical aperture), controlled using Micro-Manager 2 software. Data were acquired with a rolling shutter at 1 fps with a 200 ms exposure time. Illumination was provided by a 470-nm CoolLED system. Analysis of Ca^2+^ imaging was performed with FIJI software. Data were first converted to eight bit and processed with background subtraction, 3D smoothing and histogram-based bleach correction. If required, manual drift correction was performed. Active cells were selected and delineated from the images, guided by a maximum intensity projected image to highlight high-intensity structures. Intensity measurements were normalized as ΔF/F0, calculated as:

*100 × (fluorescence value − baseline fluorescence ÷ baseline fluorescence)*.

Baseline fluorescence was calculated as the mean intensity from 30 frames during a control period of recording. Ca^2+^ transients from astrocytes in hemisected spinal cords were detected in a semi-automated manner using Hill-Valley analysis through Dataview software (courtesy of Dr. W. J. Heitler, University of St Andrews). Events were detected using a slope height filter of 10% of the valley-to-peak height. Due to variation in Ca^2+^ event intensity and duration, false-positive events were excluded manually and some false negative events were selected manually. In hemisect experiments where events were analyzed with Dataview, Ca^2+^ event intensity was calculated from the normalized intensity, averaged across all the events within a time window for a cell and across cells of each experiment. The maximum intensity of cells is taken as the maximum normalized intensity for a cell within a time frame. Ca^2+^ images are displayed in figures as maximum projections from across 120 frames following initial processing steps.

### Whole-Cell Patch-Clamp

Whole-cell patch-clamp recordings were performed from ventral horn interneurons using glass microelectrodes (2.5–5 MΩ) filled with intracellular solution. Signals were amplified and filtered (4 kHz low-pass Bessel filter) with a Multi-Clamp 700B amplifier (Molecular Devices) and acquired at 10 kHz using a Digidata 1440A A/D board and pClamp software (Molecular Devices). Gigaseals (~2 GΩ) were obtained before the establishment of whole-cell mode and neurons with resting membrane potentials between −40 mV and −70 mV were used for experiments. All recordings were performed in current-clamp mode. Cells were given a 10 s pulse of 200 pA to induce trains of action potentials (eliciting ~100 action potentials). All experiments were conducted at ~34°C.

### Ventral Root Recording

For whole spinal cord preparations in which locomotor-related activity was induced pharmacologically, glass suction electrodes were attached to the upper lumbar segment roots (L1–3) on both the left and right side of the isolated spinal cords to assess left-right alternation and flexor-related activity. Fictive locomotion was induced with 5-HT (10 μM), NMDA (5 μM) and DA (50 μM). Preparations displaying stable fictive locomotion for ~20 min during control periods were used for full experiments with the addition of additional drugs. Data were amplified and filtered (band-pass filter 10–5,000 Hz, A-M Systems Model 1700) and acquired at a sampling frequency of 6 kHz with a Digidata 1440A analog-digital converter and Axoscope software (Molecular Devices, Sunnyvale, CA, USA). For hemisected spinal cord experiments with simultaneous Ca^2+^ imaging, only one root was attached (L1–L3). Data were amplified and filtered (30–3,000 Hz; Qjin Design) and then acquired at a sampling frequency of 6 kHz using a Digidata 1440A A/D board and AxoScope software.

### Immunohistochemistry and Fluorescence Microscopy

Spinal cords from wild type, non-transgenic C57Bl/6J mice (P6) were harvested in ice-cold recording aCSF and subsequently fixed in paraformaldehyde (4%) for 4 h at 4°C. Cords were then sunk in a 33% sucrose solution overnight and cryo-embedded for cryosectioning at 20 μm thickness. Frozen sections were washed in 0.1 M phosphate-buffered saline (PBS) and then blocked and permeabilized in 5% Bovine serum albumin (BSA) and 0.2% Triton X100 for 90 min at room temperature. Sections were incubated for 48 h at 4°C with a primary antibody solution of 2.5% BSA and 0.1% Triton X100 with rabbit polyclonal anti-mGluR5 (Abcam) and mouse monoclonal anti-glutamine synthetase (Abcam), both at 1:200 dilution. Sections were then washed five times with PBS before a secondary antibody incubation in 0.1% Triton X100 for 60 min at room temperature. Following another five washes in PBS, sections were washed once briefly in DI-H_2_O and incubated in DAPI (diluted 1:5,000 in deionized-H_2_O) for 5 min before mounting in vectashield. Fixed, stained samples were imaged at 63× with a Zeiss Axio Imager M2 microscope equipped with an Apotome.2. Illumination was provided by an HXP120 lamp, and images acquired using an MRm digital camera. Three-channel images of glutamine synthetase, mGluR5 and DAPI were captured with exposure times of 500, 100 and 30 ms, respectively. Z-stacks were acquired with all channels per slice and an optimal z-step of 0.28 μm. Images were processed and visualized in FIJI (ImageJ).

### Data Handling and Statistics

All data were stored using a 20 TB NAS drive, with hot-swappable hard drives and a back-up NAS drive (QNAP Turbo). Data files and graphs were handled and produced in Excel (Microsoft). Statistics were performed with SPSS v24 and SPSS v25 (IBM). Shapiro Wilks tests were performed to assess normality. Fictive locomotor experiments were analyzed using repeated-measures ANOVAs with a *post hoc* Bonferroni test for pairwise comparisons. Where appropriate, sphericity was assessed with Mauchly’s test and Greenhouse-Giesser corrections were applied. For Ca^2+^ imaging data where two or more groups were compared, one-way ANOVAs with *post hoc* Tukey’s test for pairwise comparisons were performed. For Ca^2+^ imaging with two groups, two-sample *T*-tests were performed. Significance is given as *p < 0.05, **p < 0.01, ***p < 0.001 and not significant (ns). Ca^2+^ traces from astrocytes are displayed with a mean smoothing over five frames. Bar charts display mean with standard error bars. Samples sizes (*n*) are stated in the results where appropriate.

## Results

### Spinal Cord Astrocytes Respond to Neuronal Activity

**Figure 1:**
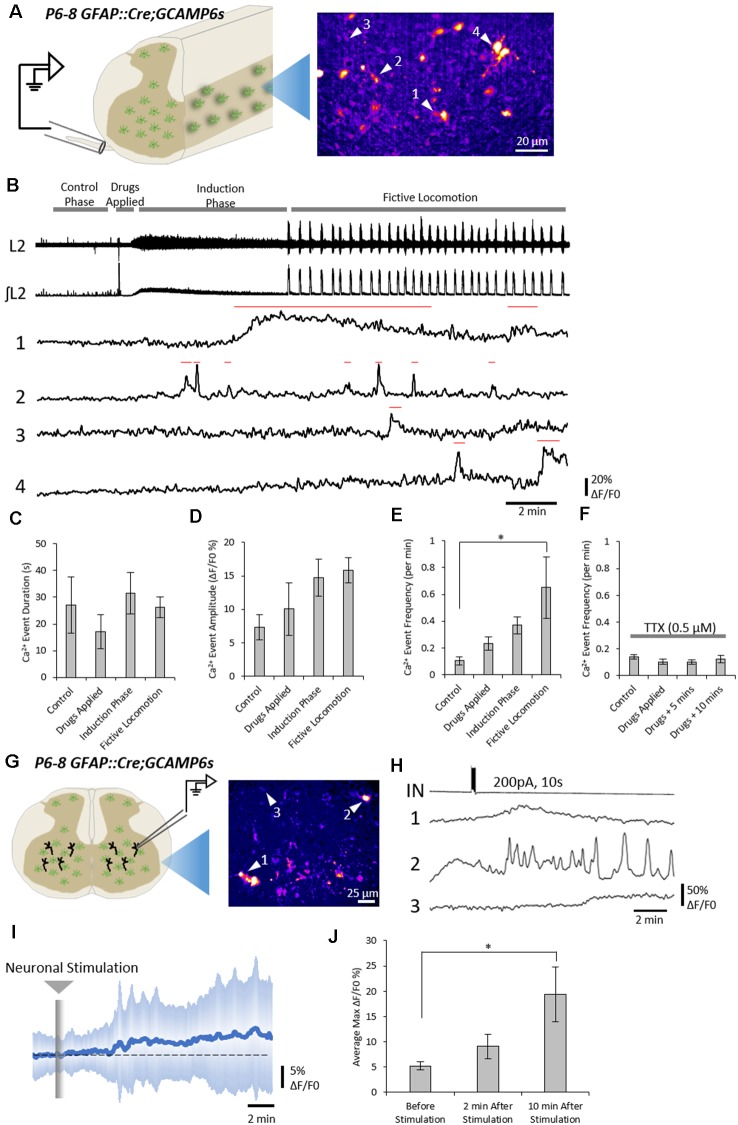
Neuronal activity evokes Ca^2+^ responses in spinal astrocytes. **(A)** Schematic of hemisected spinal cord preparations in which simultaneous ventral root recording and Ca^2+^ imaging of astrocytic activity were performed. **(B)** Example data from one experiment showing ventral root activity from baseline to the induction of fictive locomotion and Ca^2+^ imaging traces from four astrocytes (denoted in panel **A**). Red lines above traces indicate the Ca^2+^ events detected for analysis. **(C)** Bar chart showing the duration of astrocytic Ca^2+^ transients during different phases of the experiment. **(D)** Bar chart showing the intensity of astrocytic Ca^2+^ transients during different phases of the experiment. **(E)** Bar chart showing the frequency of astrocytic Ca^2+^ transients during different phases of the experiment. **(F)** Bar chart showing the frequency of astrocytic Ca^2+^ transients during different phases of the experiment when the hemisected cord was perfused with tetrodotoxin (TTX). **(G)** Schematic of a spinal cord slice with a simultaneous whole-cell patch-clamp recording from a ventral horn interneuron and Ca^2+^ imaging of astrocytic activity. **(H)** Example data from one experiment showing the activation of a spinal cord interneuron (IN), and the Ca^2+^ transients from three astrocytes (denoted in panel **G**). **(I)** Averaged Ca^2+^ imaging trace from 52 astrocytes from eight separate experiments in response to neuronal stimulation. **(J)** Bar chart showing the maximum intensity of Ca^2+^ transients in astrocytes before and after neuronal stimulation. *p < 0.05.

We first asked whether spinal astrocytes were active during fictive locomotion. Hemisected spinal cords were prepared from neonatal (P6–8) GFAP::Cre;GCAMP6s mice to enable the visualization of gray matter astrocytes, broadly in laminae VII, VIII and X, whilst simultaneously recording fictive locomotor output *via* lumbar segment roots (L1–3; depicted in the schematic of Figure 1A).

Across all experiments (108 astrocytes from *n* = 6 hemisected spinal cords), the average density of astrocytes displaying Ca^2+^ transients, as indicated by changes in fluorescence, was 400 cells per mm^2^ (ranging from 80 to 690 cells per mm^2^ between experiments). Astrocytes showed little to no activity during the “control” phase of recording, with an average frequency of 0.1 transients per minute (Figure 1B). After 3–4 min of control recording, DA (50 μM), 5-HT (10 μM) and NMDA (5 μM) were perfused to evoke fictive locomotion. Within 1–2 min of drug perfusion, a tonic increase in motor neuron activity was observed, as evidenced by an increase in the baseline of ventral root recordings, which lasted approximately 5 min. This tonic activity was followed by rhythmic locomotor-related bursts which first appeared 6–10 min after the perfusion of the locomotor drugs. Astrocytic Ca^2+^ transients showed no significant change in their duration (Figure 1C; *F*_(3)_ = 0.63; *p* = 0.602) or intensity (Figure 1D; *F*_(3)_ = 2.1; *p* = 0.133) during these different phases of ventral root output. There was also no significant change in the frequency of astrocytic Ca^2+^ transients immediately following the addition of the locomotor drugs nor during the induction phase when tonic ventral root activity was recorded. However, the frequency of astrocytic Ca^2+^ transients significantly increased above baseline levels during the period when rhythmic, locomotor-related bursting was present (Figure 1E; *F*_(3)_ = 3.6; *p* = 0.032). There was no specific coupling between individual astrocytic Ca^2+^ transients and single fictive locomotor bursts, perhaps due to the fact that the locomotor bursts recorded from ventral roots averaged 4 s in duration, while astrocytic Ca^2+^ transients averaged 25 s in duration. Nevertheless, there was a significant increase in the occurrence of astrocytic Ca^2+^ transients when fictive locomotor network activity was established.

We next confirmed that elevations in astrocytic activity were due to locomotor network activity, rather than a direct astrocytic response to the drugs used to induce locomotor-related output. We performed a series of hemisected spinal cord experiments in the presence of (TTX, 0.5 μM) to block neuronal activity (Figure 1F). As expected, no motor output was evoked when DA, 5-HT, and NMDA were applied in the presence of TTX. Analysis of astrocytic activity in these experiments (89 astrocytes from *n* = 6 hemisected spinal cords), revealed no significant change in the duration (*F*_(3)_ = 1.2; *p* = 0.326), intensity (*F*_(3)_ = 1.3; *p* = 0.291) or the frequency (Figure 1F; *F*_(3)_ = 0.78; *p* = 0.516) of Ca^2+^ transients after the addition of the locomotion-inducing drugs. These results suggest that spinal cord astrocytes are responsive to neuronal activity in the spinal cord, rather than any direct actions of the drugs used to induce locomotor-related activity.

We next asked whether spinal cord astrocytes could respond directly to interneurons within the spinal cord. To test this, we used whole-cell patch-clamp electrophysiology to evoke trains of action potentials in individual interneurons in spinal cord slices from GFAP::Cre;GCAMP6s mice (Figure 1G). A total of nine neurons were patched and stimulated. In eight out the nine experiments, we observed Ca^2+^ responses in astrocyte somas following neural stimulation (measurements from 53 astrocytes from *n* = 8 slice experiments; Figures 1H,I). The maximum intensity (ΔF/F0), averaged from responsive astrocytes within individual experiments, rose by 19.4 ± 15.3% following neuronal stimulation (Figure 1J; *F*_(2)_ = 4.4; *p* = 0.024).

### Astrocytic mGluR5 Receptors Mediate Neuron-to-Astrocyte Signaling

**Figure 2:**
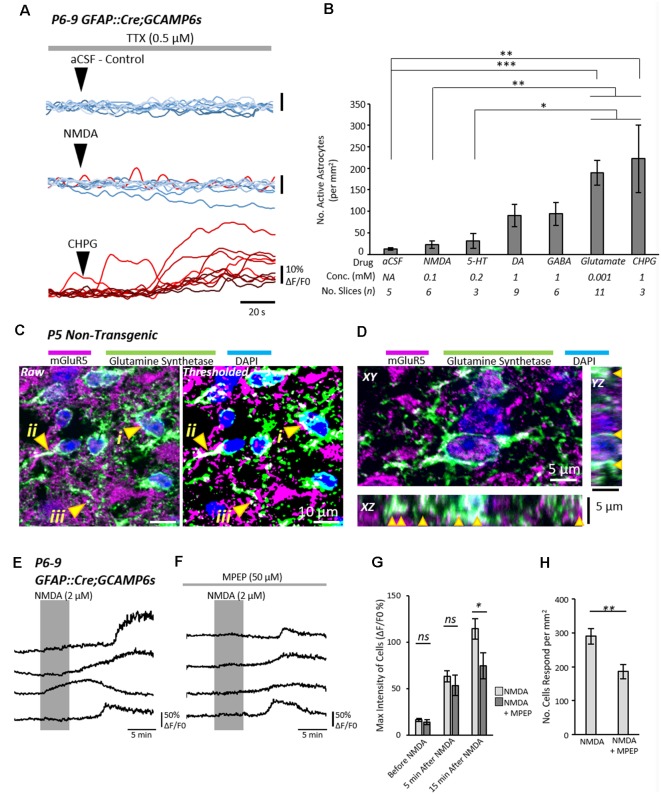
Astrocytic metabotropic glutamate receptor 5 (mGluR5) receptors mediate neuronal-to-astrocyte signaling in the mammalian spinal cord. **(A)** Example traces from Ca^2+^ imaging of astrocytes during local applications of vehicle control artificial cerebrospinal fluid (aCSF), N-methyl-D-aspartate (NMDA) and (RS)-2-Chloro-5-hydroxyphenylglycine (CHPG) in the presence of TTX (1 μM). Traces from 10 cells from within the same experiment are shown for each agonist overlayed. Traces are displayed in a red-hue if responsive to the drug, and blue if non-responsive. **(B)** Bar chart displaying the density of astrocytes that responded to a given pharmacological stimulus. The agonist concentrations and the number of slices (*n* number) for each agent are listed below the graph. **(C)** Visualization of mGluR5 receptor expression in glutamine synthetase-labeled astrocytic cell bodies (i), primary leaflet/processes (ii) and finer processes (iii). **(D)** 3D visualization of mGluR5 localization on astrocytes with orthogonal views of XY, XZ and YZ. Arrows denote cases of colocalization between mGluR5 and glutamine synthetase observed in 3D. **(E)** Application of NMDA in spinal cord slices from GFAP::Cre;GCAMP6s mice enables the visualization of astrocyte activity in response to neuronal activation. NMDA does not activate astrocytes directly (see Figure 3). **(F)** Application of NMDA in the presence of mGluR5 antagonist Methyl-6-(phenylethynyl)pyridine hydrochloride (MPEP) shows attenuated responses from astrocytes. **(G)** Bar chart showing that the degree of astrocytic activation 15 min after NMDA application was significantly reduced when slices were incubated in the mGluR5 antagonist, MPEP. **(H)** Bar chart showing the number of astrocytes that responded to neuronal activity induced by NMDA was reduced in the presence of MPEP. *p < 0.05, **p < 0.01, ***p < 0.001; ns, not significant.

Next, we sought to address how neurons were signaling to astrocytes in the spinal cord. Spinal cord slices were prepared from neonatal (P6–10) GFAP::Cre;GCAMP6s mice and astrocytic activity was visualized while receptor agonists were locally applied to the recording chamber to evoke responses from astrocytes (Figure 2A). Experiments were conducted in the presence of TTX (1 μM) to block neuronal activity. Positive responses from astrocytes within an experiment were defined as an increase in normalized fluorescence intensity (ΔF/F0) of at least 5% occurring within 2 min of agonist application (Figures 2A,B).

Glutamate and the metabotropic glutamate receptor 5 (mGluR5) agonist, CHPG, both elicited strong Ca^2+^ responses from large numbers of astrocytes, significantly more so than the vehicle control (aCSF; CHPG: *p* = 0.003; Glutamate: *p* = 0.0002), 5-HT (CHPG: *p* = 0.007; Glutamate: *p* = 0.001) and NMDA (CHPG: *p* = 0.04; Glutamate: *p* = 0.029). GABA and DA also evoked moderate responses from astrocytes, though the number of astrocytes that responded was far less than the number that responded to Glutamatergic signaling, and not significantly greater than control. Based on cell density measurements from a TdTomato fluorescence reporter (*n* = 12 slices from two individual GFAP::Cre;TdTomato mice aged P8–9; Supplementary Figure S1), we estimate that approximately 70–100% of astrocytes are activated by Glutamate and CHPG. From these results, we conclude that glutamatergic signaling *via* metabotropic receptors is the most probable mechanism by which spinal neurons communicate with astrocytes.

Next, we sought to confirm whether spinal astrocytes express the mGluR5 receptor. Immunolabeling experiments revealed widespread expression of the mGluR5 receptor in the neonatal spinal cord (Figures 2C,D). Co-labeling with the astrocytic marker, Glutamine Synthetase, confirmed that mGluR5 is expressed on the soma, primary branches and smaller branchlets of astrocytes (Figures 2C,D, arrows).

Finally, we examined whether the mGluR5 antagonist, MPEP, could reduce neuron-to-astrocyte communication. Having previously shown that astrocytes do not respond to NMDA directly in the presence of TTX, we applied NMDA to spinal cord slices for 5 min to evoke neuronal activity while visualizing astrocyte Ca^2+^ activity (221 astrocytes from *n* = 7 slice experiments). Any astrocytic responses were, therefore, the indirect result of enhanced neuronal activity induced by NMDA (Figure 2E). In a second condition (124 astrocytes from *n* = 6 slice experiments), slices were incubated in MPEP (50 μM) 15–20 min prior to, and during, NMDA application (Figure 2F). Analysis of Ca^2+^ imaging data revealed that MPEP reduced both the degree of astrocytic activation, as determined by the maximum intensity (Figure 2G; *t* = 2.4; *p* = 0.038), and the number of activated astrocytes (Figure 2H; *t* = 3.2; *p* = 0.008).

### Pharmacogenetic Manipulation of Astrocytic Activity Modulates Fictive Locomotion

Finally, we wanted to determine whether manipulating neuronal-astrocyte interactions would modulate the locomotor CPG output. In previous studies, we have used pharmacological methods to excite spinal astrocytes (Acton and Miles, 2015; Acton et al., 2018). Here we decided to employ pharmacogenetics to more specifically excite and inhibit astrocytes, an approach that has been used by others to manipulate astrocytic activity (Yang et al., 2015). Transgenic DREADD (Designer Receptor Exclusively Activated by Designer Drug) mice were crossed with GFAP::Cre mice to produce GFAP::Cre;hM3Dq and GFAP::Cre;hM4Di offspring. In spinal cords or spinal slices from GFAP::Cre;hM3Dq and GFAP::Cre;hM4Di neonates, perfusion with the specific DREADD agonist Clozapine-N-Oxide (CNO) should excite (hM3Dq) or inhibit (hM4Di) astrocytes.

**Figure 3:**
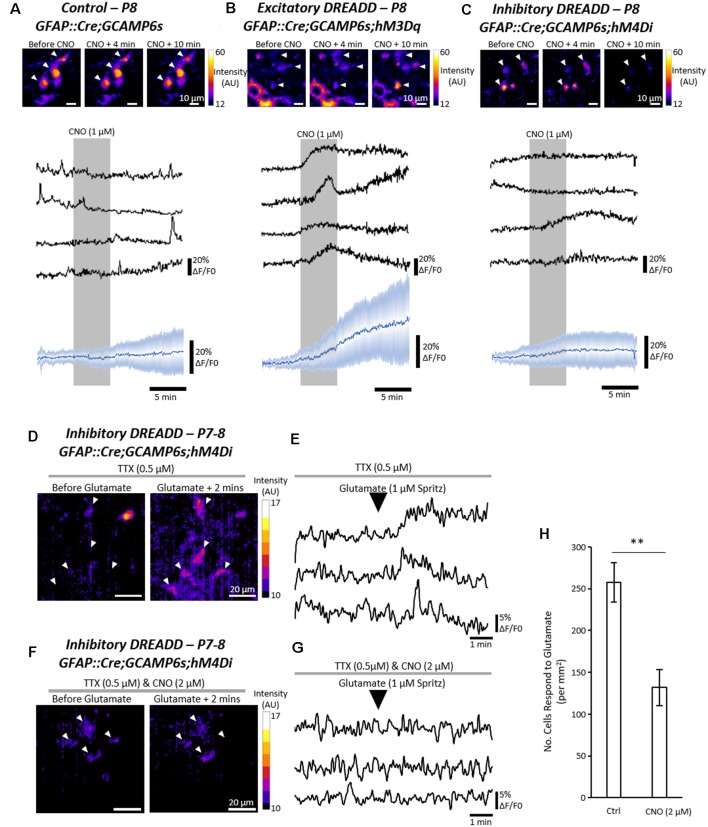
Pharmacogenetic techniques can manipulate astrocytic Ca^2+^ activity in the mammalian spinal cord. **(A)** Ca^2+^ imaging of GFAP::cre;GCAMP6s astrocytes in response to Clozapine-N-Oxide (CNO). Example (top), and averaged (bottom) traces of Ca^2+^ imaging from astrocytes during applications of CNO (19 cells for averaged trace). **(B)** Ca^2+^ imaging of GFAP::cre;GCAMP6s;hM3Dq astrocytes in response to CNO. Example (top), and averaged (bottom) traces of Ca^2+^ imaging from astrocytes during applications of CNO (25 cells for averaged trace). **(C)** Ca^2+^ imaging of GFAP::cre;GCAMP6s;hM4Di astrocytes in response to CNO. Example (top), and averaged (bottom) traces of Ca^2+^ imaging from astrocytes during applications of CNO (30 cells for averaged trace). **(D)** Ca^2+^ responses in GFAP::cre;GCAMP6s;hM4Di astrocytes in response to glutamate, perfused in TTX to prevent neuronal-mediated responses. **(E)** Example traces of Ca^2+^ responses in GFAP::cre;GCAMP6s;hM4Di astrocytes in response to glutamate. **(F)** Ca^2+^ responses in GFAP::cre;GCAMP6s;hM4Di astrocytes in response to glutamate, perfused in both TTX and CNO to inhibit astrocyte activity. **(G)** Example traces of Ca^2+^ responses in GFAP::cre;GCAMP6s;hM4Di astrocytes showing no response to glutamate due to pharmacogenetic inhibition with CNO. **(H)** Bar chart displaying the number of astrocytes responding to glutamate in GFAP::cre;GCAMP6s;hM4Di spinal cord slices under control conditions and when perfused with CNO to inhibit astrocytes. **p < 0.01.

To first validate the ability of the DREADD receptors to excite or inhibit astrocytes in hM3Dq and hM4Di mice respectively, Ca^2+^ imaging was performed in triple transgenic mice expressing GFAP::Cre;GCAMP6s;hM3Dq or GFAP::Cre;GCAMP6s;hM4Di (Figures 3A–C). Perfusion of CNO (1 μM) to spinal cord slices evoked robust Ca^2+^ responses in astrocytes expressing hM3Dq (Figure 3A) but had no clear effect on non-transgenic control astrocytes (Figure 3B) or astrocytes expressing hM4Di (Figure 3C). We then confirmed that activation of the inhibitory hM4Di receptor with CNO (2 μM) diminished the responses of astrocytes to applications of glutamate, as compared with hM4Di-expressing astrocytes that were not incubated in CNO (Figures 3D–H; *n* = 4 slices; *t* = 4.0; *p* < 0.007). Similarly, we demonstrated that 26 astrocytes from the same GFAP::Cre;GCAMP6s;hM4Di spinal cord slice showed little response to glutamate in the presence of CNO (2 μM), but showed more typical responses to glutamate after the CNO was washed off (Supplementary Figure S2). These results suggest that pharmacogenetics can be used to either excite or inhibit spinal cord astrocytes using Cre-driven hM3Dq and hM4Di receptor activation respectively.

**Figure 4:**
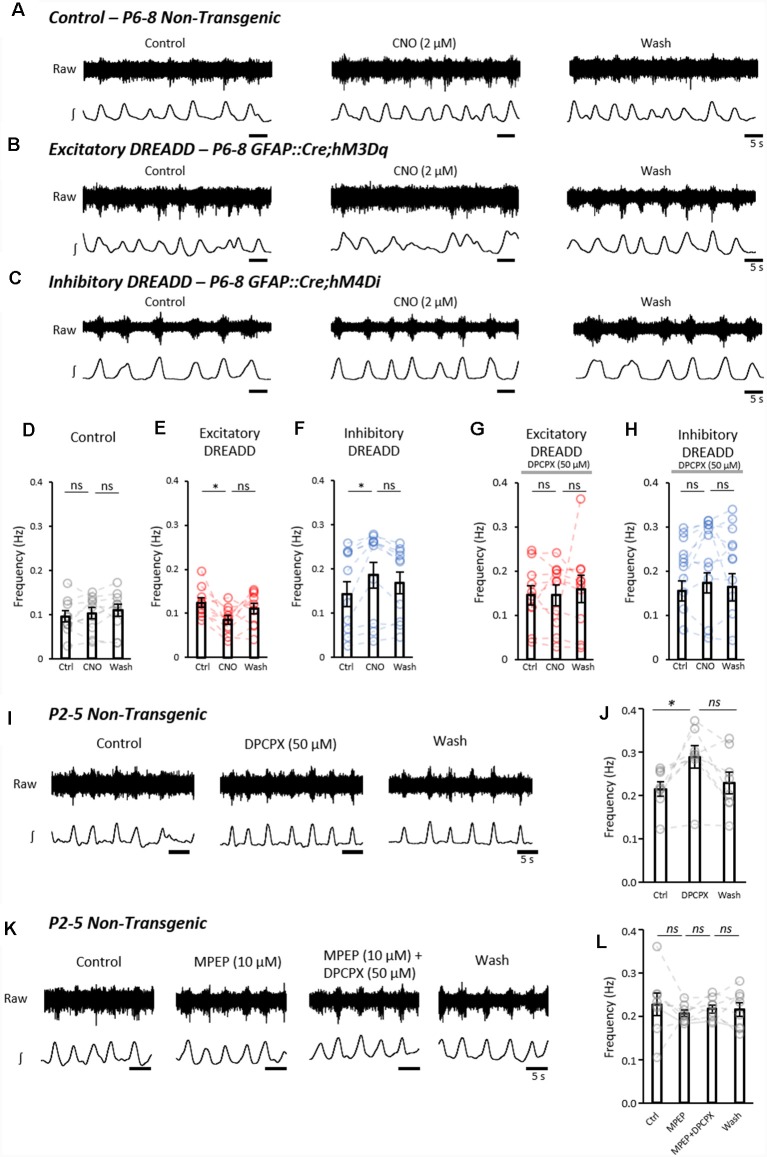
Astrocytes exert purinergic modulation of locomotion in response to mGluR5 dependent activation. **(A)** Example raw (top) and rectified/integrated (bottom) traces of pharmacologically induced fictive locomotor bursts recorded from *in vitro* spinal cord preparations from control non-transgenic neonatal mice. The burst frequency, duration and amplitude were analyzed in control conditions, in the presence of CNO and following the washout of CNO. **(B)** Pharmacologically induced fictive locomotor bursts recorded from a GFAP::cre;hM3Dq (excitatory DREADD) neonatal mouse spinal cord *in vitro* during control conditions, in the presence of CNO and following the washout of CNO. **(C)** Pharmacologically induced fictive locomotor bursts recorded from a GFAP::cre;hM4Di (inhibitory DREADD) neonatal mouse spinal cord *in vitro* during control conditions, in the presence of CNO and following the washout of CNO. **(D)** Bar chart displaying the frequency of fictive locomotor bursts in control, non-transgenic mouse spinal cords in response to CNO. **(E)** Bar chart displaying the frequency of fictive locomotor bursts in excitatory DREADD mouse spinal cords in response to CNO. **(F)** Bar chart displaying the frequency of fictive locomotor bursts in inhibitory DREADD mouse spinal cords in response to CNO. **(G)** Bar chart displaying the frequency of fictive locomotor bursts in excitatory DREADD mouse spinal cords in response to CNO when perfused with 8-Cyclopentyl-1,3-dipropylxanthine (DPCPX) throughout to block the adenosine A1 receptor. **(H)** Bar chart displaying the frequency of fictive locomotor bursts in inhibitory DREADD mouse spinal cords in response to CNO when perfused with DPCPX throughout to block the adenosine A1 receptor. **(I)** Example traces of fictive locomotion recorded from *in vitro* spinal cord preparations from control non-transgenic neonatal mouse spinal cords, showing the effects of a 20 min application of the A1 receptor antagonist, DPCPX. **(J)** Bar chart showing that the frequency of fictive locomotion is significantly increased with the application of DPCPX. **(K)** Example traces of fictive locomotion recorded from *in vitro* spinal cord preparations, showing blockade of the effects of the application of DPCPX when in the presence of MPEP. **(L)** Bar chart showing that DPCPX has no effect on the frequency of fictive locomotion when spinal cords are also incubated in MPEP. *p < 0.05, ns; not significant.

We next addressed whether activation or inhibition of astrocytes using DREADDs could modulate fictive locomotion recorded from isolated spinal cord preparations. Fictive locomotion was recorded from the upper lumbar roots (L1–3) of isolated spinal cords from control (*n* = 9), GFAP::Cre;hM3Dq (hM3Dq; *n* = 10) and GFAP::Cre;hM4Di (hM4Di; *n* = 11) mice (Figures 4A–C). There was no significant difference in the baseline amplitude, duration or frequency of locomotor bursts between control, hM3Dq and hM4Di cords (SupplementaryFigures S3A,B), confirming that the expression of the DREADD receptors in astrocytes alone had no significant effect on spinal motor circuits. The application of CNO (2 μM) had no effect on the frequency of fictive locomotion in control spinal cords (Figure 4D; *F*_(2,18)_ = 1.5; *p* = 0.256). However, CNO caused a significant reduction in burst frequency in hM3Dq cords (Figure 4E; *F*_(2,18)_ = 4.0; *p* < 0.05). In contrast, CNO elicited an increase in burst frequency in hM4Di cords (Figure 4F; *F*_(2,20)_ = 9.5; *p* < 0.01). CNO had no significant effect on burst duration (Supplementary Figures S3C–E) or amplitude (Supplementary Figures S3F–H) in control, hM3Dq or hM4Di cords.

It has previously been suggested that pharmacologically stimulated astrocytes modulate fictive locomotion through adenosine signaling (Acton and Miles, 2015; Witts et al., 2015; Acton et al., 2018), which can be inhibited by the A1 adenosine receptor antagonist, DPCPX at 50 μM (Witts et al., 2012; Acton and Miles, 2015). We, therefore, utilized DPCPX to assess whether modulation of fictive locomotion following pharmacogenetic manipulation of astrocytic activity involves adenosine signaling. We found that there was no change in locomotor frequency when astrocytes in hM3Dq (*n* = 10) and hM4Di (*n* = 11) cords were stimulated with CNO in the presence of DPCPX (50 μM; hM3Dq: *F*_(1.28,11.5)_ = 0.23; *p* = 0.702; hM4Di: *F*_(2,20)_ = 1.0; *p* = 0.414; Figures 4G,H).

Finally, we interrogated the neuron-astrocyte signaling mechanism responsible for driving astrocyte-derived purinergic modulation of the locomotor CPG. Having shown that glutamate and the mGluR5 agonist, CHPG, evoked robust Ca^2+^ responses in a high proportion of spinal astrocytes, we hypothesized that glutamatergic signaling, acting *via* mGluR5, represents the physiological activator of astrocyte-derived adenosine-dependent modulation. We, therefore, assessed whether blocking the mGluR5 receptor could reduce astrocytic adenosine-mediated modulation of the locomotor CPG. In isolated spinal cords from control non-transgenic mice (P1–5; *n* = 8), fictive locomotion was induced pharmacologically, and DPCPX (50 μM) was applied for 20 min to block astrocyte-derived adenosine (Figure 4I). Consistent with the pharmacogenetic inhibition of astrocytes, A1-receptor inhibition with DPCPX significantly increased the frequency of fictive locomotion (Figure 4J; *F*_(2,14)_ = 5.9; *p* < 0.05). DPCPX showed no effect on burst duration (data not shown, *F*_(2,14)_ = 1.9; *p* = 0.19) or amplitude (data not shown, *F*_(2,14)_ = 0.9; *p* = 0.44). In separate experiments, fictive locomotion was initiated in spinal cords (*n* = 8) which were then subsequently perfused with the mGluR5 antagonist MPEP (10 μM) for 20 min, followed by MPEP and DPCPX together, after-which both MPEP and DPCPX were washed out (Figure 4K). No significant differences in the frequency of locomotion were observed between Control, MPEP, MPEP+DPCPX and Washout conditions (Figure 4L; *F*_(1.53,10.73)_ = 0.35; *p* = 0.657). From this final experiment, we conclude that inhibiting mGluR5-mediated activation of astrocytes reduces the astrocytic release of purines and thus the degree of adenosine-dependent neuromodulation of the locomotor CPG.

## Discussion

In this study, we have used pharmacogenetic manipulation of astrocytes to show that astrocytes actively modulate spinal cord motor circuitry. Furthermore, live imaging of astrocytic activity in the isolated spinal cord suggests they respond to the activity of ventral interneurons that may form part of the spinal cord locomotor CPG. The evidence we present here suggests a modulatory feedback loop between astrocytes and neurons that depends on bi-directional communication between these two cell groups. In our proposed model, locomotor CPG activity, and associated increases in glutamatergic signaling between CPG neurons, activates astrocytes through the mGluR5 receptor. Astrocytic activation evokes the release of ATP which is metabolized extracellularly to adenosine. Adenosine acts at A1 receptors to inhibit interneurons within the CPG, which in turn affects the frequency of motor output.

Previous research has focussed on the astrocyte-to-neuron communication mechanisms that may impact motor circuits (Witts et al., 2012, 2015; Acton and Miles, 2015, 2017; Acevedo et al., 2016; Acton et al., 2018; Rivera-Oliver et al., 2018). In this study, we have identified previously unexplored neuron-to-astrocyte signaling mechanisms which may drive astrocyte-dependent modulation of the spinal cord locomotor CPG. We reveal that astrocytes are active during fictive locomotion in the isolated hemisected spinal cord, supporting observations from *in vivo* imaging of glia in the awake and moving adult mouse (Sekiguchi et al., 2016). The results from our experiments utilizing pharmacologically-induced fictive locomotion in the hemisected spinal cord do not definitively distinguish whether astrocytic activity gradually increases following the pharmacological stimulation of the neurons or whether astrocytic activity is specifically enhanced when rhythmic neural activity emerges. However, we also observed astrocytic responses to direct stimulation of ventral horn interneurons, and given that astrocyte manipulation using DREADDs only affected the frequency of locomotor output, these results offer compelling evidence that astrocytic activity is functionally linked to the locomotor CPG interneurons.

We provide compelling evidence that mGluR5 is necessary for the neuron-astrocyte signaling pathway that drives astrocytic modulation within the spinal cord. There is considerable literature on the expression of mGluRs by astrocytes and the role of mGluR5 at the tripartite synapse as a mechanism for astrocytes to detect synaptic activity (Ota et al., 2013; Panatier and Robitaille, 2016). Our data supports this theory, as spinal astrocytes express mGluR5 and responded robustly to the mGluR5 agonist, CHPG. Blocking the mGluR5 receptor reduced astrocytic activity and blocked the modulatory effects of astrocyte-derived adenosine on fictive locomotion, suggesting that mGluR5 signaling could be the “driving force” behind astrocytic modulation of motor output.

We have previously shown that astrocyte activation in P1–4 neonatal mouse spinal cords using a PAR1 agonist reduces the frequency of fictive locomotion (Acton and Miles, 2015), which matches our current findings here that DREADD-based activation of astrocytes in P6–8 spinal cords leads to reduced locomotor frequency. This suggests that the neuronal-astrocytic interactions underlying this modulatory feedback mechanism are preserved throughout early developmental stages. Whether this bi-directional signaling mechanism exists in adult mice remains to be explored. On the one hand, there is evidence that spinal astrocytes respond to walking-like behavior in the awake adult mouse (Sekiguchi et al., 2016), in a manner that is comparable to the effects we observe in our study. On the other hand, mGluR5 expression in astrocytes may not exist past adolescence (Sun et al., 2013), suggesting there could be developmentally regulated alterations in this bi-directional signaling pathway controlling the locomotor CPG.

Interestingly, mGluR5 antagonism did not completely abolish the Ca^2+^ transients we observed in astrocytes in response to neuronal activity. This suggests that multiple, parallel pathways of neuron-to-astrocyte signaling may occur in the spinal cord. Several other neurotransmitters and neuromodulators evoked Ca^2+^ transients in small numbers of spinal astrocytes. Although these responses were not significantly greater than those observed after applications of aCSF, we cannot exclude the possibility that non-mGluR5 mediated neural-glial transmission may occur. The inhibitory neurotransmitters GABA and Glycine may be the most likely candidates for low-level neuron-to-astrocyte signaling, given the increasing body of literature on inhibitory tripartite synapse interactions (Garrett and Weiner, 2009; Matos et al., 2018; Allen, 2019; Nagai et al., 2019), the expression of inhibitory neurotransmitter receptors by spinal glial cells (Kirchhoff et al., 1996), and evidence that spinal astrocytes may modulate inhibitory synaptic release probability (Witts et al., 2015). While there is some evidence that astrocytes may express 5-HT and DA receptors, activation of these receptors may not lead to Ca^2+^ transients, and may instead hyperpolarize astrocytes (Hösli et al., 1987; Miyazaki et al., 2013). Although DA evoked Ca^2+^ responses in a small number of astrocytes, we believe that DA is unlikely to be a key activator of spinal astrocytes. The mammalian spinal cord lacks dopaminergic neurons; all dopaminergic input to the spinal cord is derived from descending systems. Thus, DA could not mediate the responses we observed in astrocytes following the direct stimulation of interneurons within isolated spinal cord tissue. Furthermore, astrocytes only showed elevated activity in hemisected spinal cord preparations once clear locomotor activity was established, with no significant response to the application of DA along with 5-HT and NMDA in the presence of TTX. These findings, therefore, indicate that astrocytes are responsive to intrinsic spinal neurotransmitters released during physiological network activity.

In addition to classic transmitter-based signaling, changes in the extracellular environment induced by neuronal activity may also stimulate astrocytic activity. For example, rises in extracellular K+ concentration, which occurs during neuronal activity including that of the locomotor CPG (Brocard et al., 2013), represents an alternative mechanism by which astrocytes may be activated (Scemes and Spray, 2012; Bellot-Saez et al., 2017). Given the number of different neurotransmitter receptors that are reported to be expressed by astrocytes throughout the nervous system (Verkhratsky and Butt, 2013) and the ability of spinal astrocytes to respond to multiple neuronally-derived signals, it is possible that astrocytes act as global integrators of neuronal activity and contribute to a wide range of spinal neuromodulatory systems.

There is converging evidence that stimulating astrocytes pharmacologically, or ablating astrocytes with gliotoxins, reveals purinergic-dependent, glial-derived modulation of the spinal cord locomotor CPG (Dale and Gilday, 1996; Dale, 1998; Brown and Dale, 2000; Witts et al., 2012, 2015; Acton and Miles, 2015; Acevedo et al., 2016; Acton and Miles, 2017; Acton et al., 2018; Rivera-Oliver et al., 2018). Although astrocytes in other CNS regions demonstrate the ability to utilize other transmitters, such as glutamate or GABA (Malarkey and Parpura, 2008; Christensen et al., 2018); there is a paucity of evidence to suggest that other gliotransmitters are involved in the astrocytic control of the locomotor CPG. In previous studies, antagonists of purinergic signaling were sufficient to block all the astrocytic effects on the locomotor network (Acton and Miles, 2015; Witts et al., 2015; Acton and Miles, 2017; Acton et al., 2018). In this study, we have employed the pharmacogenetic DREADD approach to specifically target astrocytes and allow us to both excite and inhibit astrocytes. The hM3Dq-based activation of astrocytes elicits a strong response comparable with the use of the PAR1 agonist, TFLLR, which has been used to excite astrocytes in previous similar studies (Acton and Miles, 2015; Acton et al., 2018). While these high levels of stimulation may invoke non-physiological responses from cells, it is important to note that the hM4Di-based inhibition of astrocytes results in a complementary, opposing effect on fictive locomotion as compared to stimulating astrocytes. Furthermore, the effects of stimulating and inhibiting astrocytes were both nullified by blocking A1 adenosine receptors. Our data, therefore, support previously proposed mechanisms of glial-mediated modulation of the spinal locomotor CPG. The theory stands that astrocytes release ATP, which is subsequently converted to adenosine, which then acts at heteromeric dimers comprised of A1 adenosine and D1 dopaminergic receptors on CPG interneurons to reduce neuronal activity (Witts et al., 2015; Acton and Miles, 2017; Acton et al., 2018; Rivera-Oliver et al., 2018).

There remains debate as to whether gliotransmission occurs under physiological conditions, or whether reported incidences of glia-to-neuron signaling are merely artifacts of *in vitro* studies or the stimulation method employed (Fiacco and McCarthy, 2018; Savtchouk and Volterra, 2018). As demonstrated by our study, the locomotor circuitry of the spinal cord represents a useful model in which to interrogate these questions. The spinal cord can be isolated for experimentation while still conserving the essential sensory inputs and motor outputs for reflex responses and locomotor activity. The outputs generated by spinal circuits are measurable and directly correlative to the *in vivo* behaviors they serve. Thus, spinal circuits may provide a practical solution to the gliotransmission debate by avoiding the caveats of other approaches such as *in vitro* cell culture studies.

Our results strongly imply a physiological role for the astrocytic release of purines in modulating a basic, neuronally-mediated behavior. What remains to be investigated is the precise gliotransmitter release mechanism in spinal astrocytes; i.e., whether transmission utilizes vesicular release machinery or occurs *via* hemichannel pores formed by connexins. This could be addressed by using mutant mice with perturbed Vesicle-Associated Membrane Proteins (VAMPs) or mutated Connexin-43 which forms hemichannel pores. Recent findings suggest reactive astrocytes in ALS display enhanced expression of Connexin 43, contributing to motor neuron toxicity (Almad et al., 2016). Therefore, understanding the precise signaling machinery between both astrocytes and neurons may reveal therapeutic targets for treating neurodegenerative diseases, such as ALS.

The locomotor CPG is just one of several rhythmic neural circuits of the CNS in which gliotransmission has been interrogated. Astrocytes have been shown to be active in the respiratory control networks of the brainstem (Guyenet et al., 2009; Gourine et al., 2010; Okada et al., 2012; Sheikhbahaei et al., 2018). Chemosensitive astrocytes in the retrotrapezoid nucleus respond to circulating CO_2_ and consequently release ATP to initiate respiration (Gourine et al., 2010; Huxtable et al., 2010; Sheikhbahaei et al., 2018). Meanwhile, the ATP metabolite, adenosine, has been shown to exhibit an inhibitory effect on the respiratory CPG (Huxtable et al., 2010). Studies into mastication have revealed an entirely different astrocytic modulatory mechanism. In the rat trigeminal sensorimotor circuit, astrocytes release the Ca^2+^ binding protein S100β, which sequesters extracellular Ca^2+^ and reduces NMDA-induced bursting of rhythmogenic neurons that lead to chewing behavior (Morquette et al., 2015). It is conceivable that this mechanism is also present in other networks such as those controlling respiration or locomotion.

Feeding behavior is also regarded as rhythmic, though not strictly under the control of a CPG but rather orchestrated by an “entrained oscillator” in the medial basal hypothalamus (Mieda et al., 2006). Here in the hypothalamus, astrocytes also act as a source of adenosine to modulate feeding behavior (Yang et al., 2015). In this instance, pharmacogenetic manipulation of astrocytes demonstrated their ability to bi-directionally modulate food intake in mice, helping to maintain feeding within a constrained range to prevent energy deficit or surfeit.

Glial cells have also been implicated in the control of rhythmic CPGs in the periphery, for example, within networks of the enteric nervous system that regulate gut motility patterns (Smith and Koh, 2017). Here it has been shown that enteric glia (likened to CNS astrocytes) are responsive to neural-mediated motor events that aid in colonic transport of fecal matter (MacEachern et al., 2011; Broadhead et al., 2012; McClain et al., 2014, 2015). Genetic manipulations that perturb astrocytic gliotransmitter release mechanisms significantly affected colonic fecal transit in mice (Grubišić and Parpura, 2017). The nature of the enteric glial transmitter involved remains unknown, but both ATP and NO are implicated (Gulbransen and Sharkey, 2012).

There are clearly diverse roles for astrocytes and other glial cells throughout the nervous system. A growing body of evidence indicates that astrocytes play active roles within many neural circuits, as they are able to respond to neuronal signaling and release chemical messengers *via* gliotransmission. While it remains to be determined whether there is any conserved logic to the role of astrocytes within neural circuits, our study on the locomotor CPG indicates a purinergic-dependent modulation of rhythmogenic neural network activity, which is driven by glutamate derived from the neural network itself. Based on previous literature (Yang et al., 2015; Cinelli et al., 2017; Sheikhbahaei et al., 2018), it is conceivable that astrocyte-derived purinergic modulation is a key mechanism within rhythmic circuits, which helps to constrain network output in order to control rhythmic behaviors. Further advances in our knowledge of the roles of glial cells within neural circuits will pave the way for a greater understanding of how the nervous system coordinates behavior.

## Data Availability Statement

The datasets generated for this study are available on request to the corresponding author.

## Ethics Statement

The animal study was reviewed and approved by the University of St Andrews Animal Welfare and Ethics Committee and conducted in accordance with the UK Animals (Scientific Procedures) Act 1986.

## Author Contributions

MB designed and performed the experiments, data analysis and wrote the manuscript. GM designed the experiments, wrote the manuscript and supervised the project.

## Conflict of Interest

The authors declare that the research was conducted in the absence of any commercial or financial relationships that could be construed as a potential conflict of interest.

## Supplementary Material

FIGURE S1 Astrocyte cell density using a fluorescence reporter line. **(A)** Spinal cord slices were prepared from GFAP::Cre;TdTomato neonatal mice and images of the ventral horn were captured. Red fluorescent cells were counted to estimate the total density of astrocytes (*n* = 12 spinal cord slices from two mice, P8–9). **(B)** Box and whisker plot showing the average number of TdTomato +ve astrocytes in neonatal spinal cord slices per mm^2^.

FIGURE S2 Inhibition of spinal astrocytes using hM4Di DREADD receptor activation. **(A)** Spinal cord slice prepared from a neonatal P8 GFAP::Cre;GCAMP6s;hM4Di mouse. Astrocytes from the same slice were visualized using Ca^2+^ imaging to investigate their responses to Glutamate with and without the presence of CNO to inhibit them (*n* = 26). **(B)** Astrocytes were first visualized in the presence of TTX and CNO, to block neurons and hM4Di-expressing astrocytes respectively. Three example traces are shown of three astrocytes, and an averaged trace of the ΔF/F0 with standard deviation from a total of 26 astrocytes. **(C)** After the first recording, CNO was washed off for 15 min leaving only TTX to inhibit the neurons. The three traces depict the ΔF/F0 from the same three cells as in panels **(A,B)**. A greater number of cells responded to glutamate, typically showing a more sustained ΔF/F0 elevation, when CNO was absent.

FIGURE S3 Additional fictive locomotor bursting parameters for control (non-transgenic), GFAP::cre;hM3Dq and GFAP::cre;hM4Di neonatal spinal cords. **(A)** Bar chart showing the duration of fictive locomotor bursts in each of the three genotypes of mice used. **(B)** Bar chart showing the frequency of fictive locomotor bursts in each of the three genotypes of mice used. **(C)** Bar charts displaying the burst duration under control, CNO and wash conditions for control (non-transgenic) spinal cords. **(D)** Bar charts displaying the burst duration under control, CNO and wash conditions for GFAP::Cre;hM3Dq spinal cords. **(E)** Bar charts displaying the burst duration under control, CNO and wash conditions for GFAP::Cre;hM4Di spinal cords. **(F)** Bar charts displaying the burst amplitude under control, CNO and wash conditions for control (non-transgenic) spinal cords. **(G)** Bar charts displaying the burst amplitude under control, CNO and wash conditions for GFAP::Cre;hM3Dq spinal cords. **(H)** Bar charts displaying the burst amplitude under control, CNO and wash conditions for GFAP::Cre;hM4Di spinal cords. ns, not significant.
