# Supplementary figures and images for: Bi-Directional Communication Between Neurons and Astrocytes Modulates Spinal Motor Circuits
Source: Front Cell Neurosci. 2020 Feb 27;14:30. doi: 10.3389/fncel.2020.00030 (PMC7057799; doi:10.3389/fncel.2020.00030)

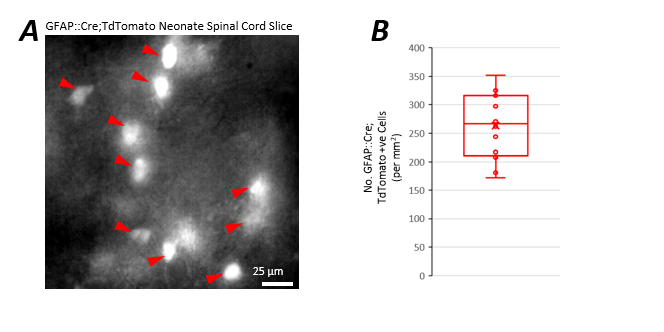

Supplement: FIGURE S1 — Astrocyte cell density using a fluorescence reporter line. (A) Spinal cord slices were prepared from GFAP::Cre;TdTomato neonatal mice and images of the ventral horn were captured. Red fluorescent cells were counted to estimate the total density of astrocytes (n = 12 spinal cord slices from two mice, P8–9). (B) Box and whisker plot showing the average number of TdTomato +ve astrocytes in neonatal spinal cord slices per mm2. [file Image_1.TIF]

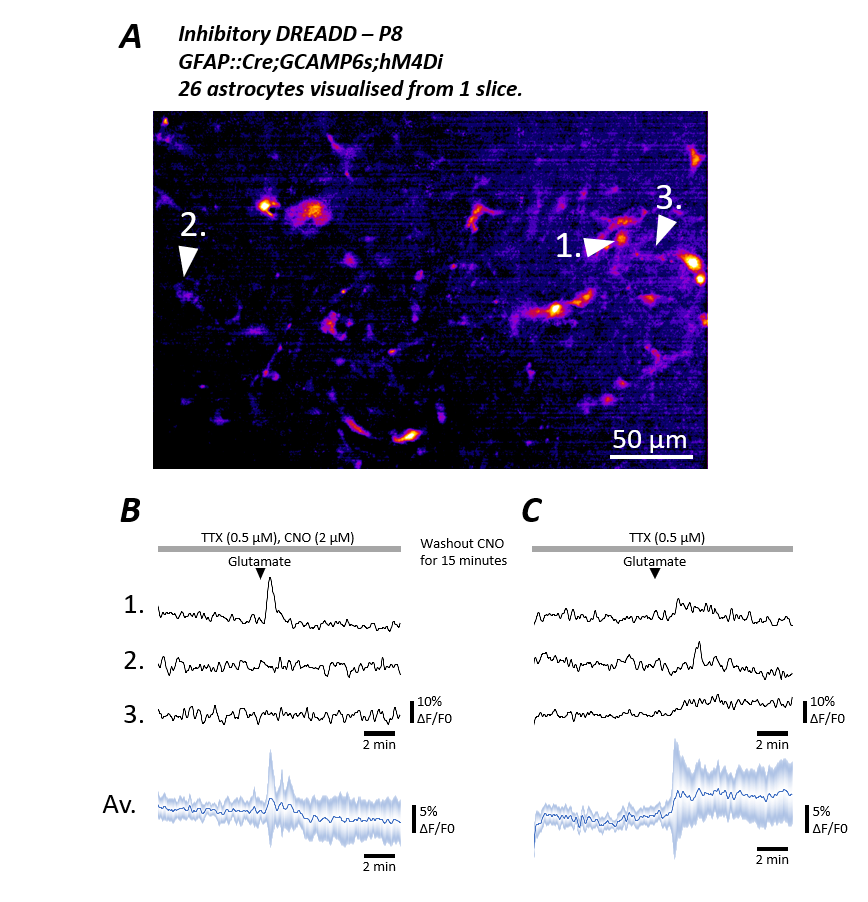

Supplement: FIGURE S2 — Inhibition of spinal astrocytes using hM4Di DREADD receptor activation. (A) Spinal cord slice prepared from a neonatal P8 GFAP::Cre;GCAMP6s;hM4Di mouse. Astrocytes from the same slice were visualized using Ca2+ imaging to investigate their responses to Glutamate with and without the presence of CNO to inhibit them (n = 26). (B) Astrocytes were first visualized in the presence of TTX and CNO, to block neurons and hM4Di-expressing astrocytes respectively. Three example traces are shown of three astrocytes, and an averaged trace of the ΔF/F0 with standard deviation from a total of 26 astrocytes. (C) After the first recording, CNO was washed off for 15 min leaving only TTX to inhibit the neurons. The three traces depict the ΔF/F0 from the same three cells as in panels (A,B). A greater number of cells responded to glutamate, typically showing a more sustained ΔF/F0 elevation, when CNO was absent. [file Image_2.TIF]

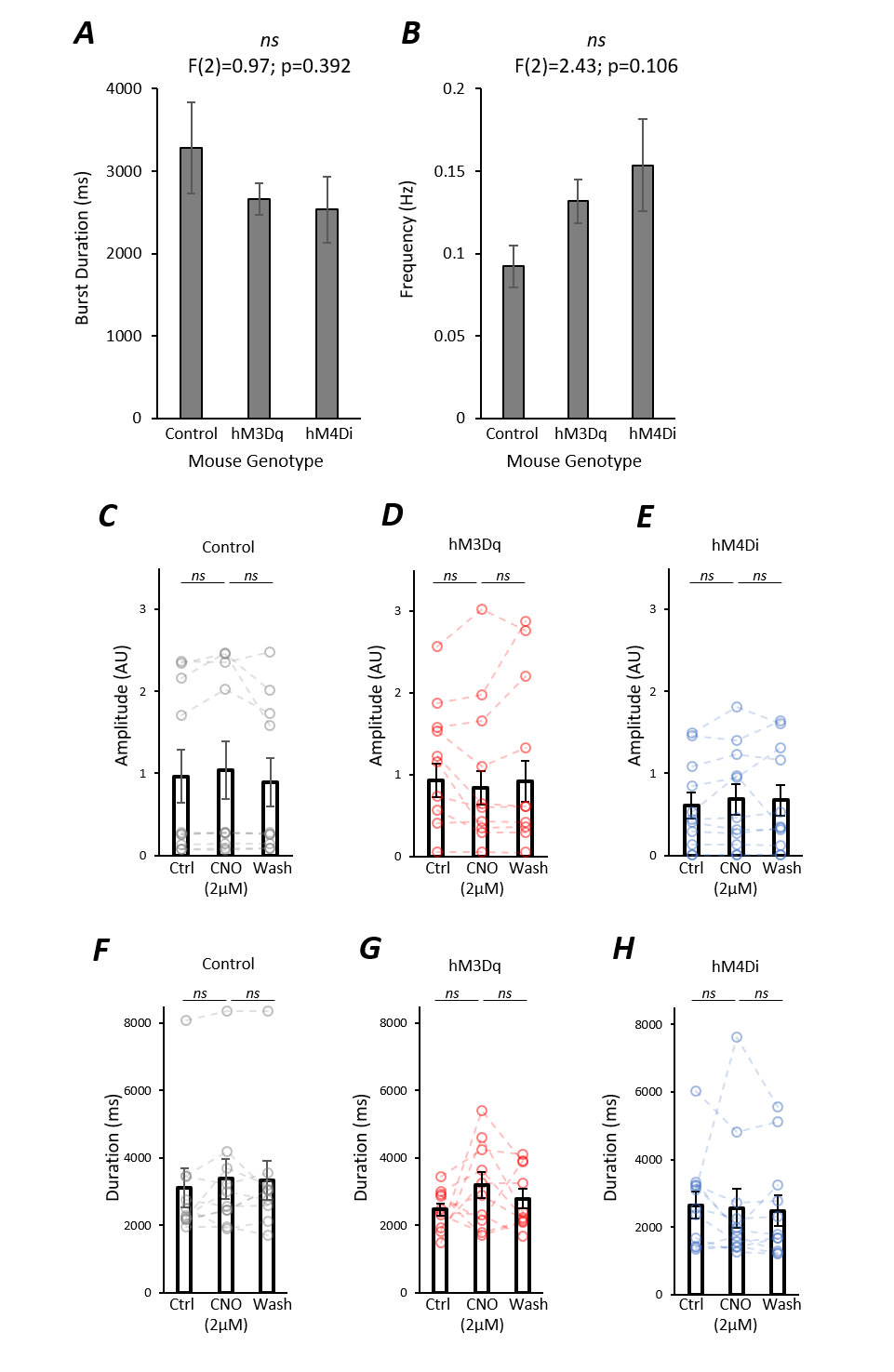

Supplement: FIGURE S3 — Additional fictive locomotor bursting parameters for control (non-transgenic), GFAP::cre;hM3Dq and GFAP::cre;hM4Di neonatal spinal cords. (A) Bar chart showing the duration of fictive locomotor bursts in each of the three genotypes of mice used. (B) Bar chart showing the frequency of fictive locomotor bursts in each of the three genotypes of mice used. (C) Bar charts displaying the burst duration under control, CNO and wash conditions for control (non-transgenic) spinal cords. (D) Bar charts displaying the burst duration under control, CNO and wash conditions for GFAP::Cre;hM3Dq spinal cords. (E) Bar charts displaying the burst duration under control, CNO and wash conditions for GFAP::Cre;hM4Di spinal cords. (F) Bar charts displaying the burst amplitude under control, CNO and wash conditions for control (non-transgenic) spinal cords. (G) Bar charts displaying the burst amplitude under control, CNO and wash conditions for GFAP::Cre;hM3Dq spinal cords. (H) Bar charts displaying the burst amplitude under control, CNO and wash conditions for GFAP::Cre;hM4Di spinal cords. ns, not significant. [file Image_3.TIF]
